# Do we need a change in transfusion strategy in patients with subarachnoidal hemorrage?

**DOI:** 10.1186/2197-425X-3-S1-A772

**Published:** 2015-10-01

**Authors:** S Valles Angulo, MP Gracia Arnillas, I Dot Jordana, E Cuadrado Godia, A Ois Santiago, F Vasco Castaño, R Muñoz Bermudez, M Samper Sanchez, E Vivas Diaz, G Villalba Martinez

**Affiliations:** Hospital del Mar, Barcelona, Spain

## Objective

To assay at what hemoglobin (Hb) levels patients recieved transfusion support, and the impact that this may have in prognosis and possible adverse effects in patients admitted with aneurismatic subarachnoidal hemorrage.

## Methods

This is a retrospective observational study, including all patients admitted in our hospital diagnosed of not-traumatic SAH, both in the Intensive Care Unit and in Stroke Unit, between 2007 and 2014. Demographics, cardiovascular risk factors, neurological deficit, GCS, Hunt & Hess and Fisher scales, bleeding localization, aneurysm, incidence of complications (hydrocephalus, intracraneal hipertension, stroke, vasospasm), treatment (surgical or endovascular) as well as mortality were collected. We analyzed hemoglobin levels during the admittance, dividing the patients in 3 categories: Hb < 7 g/dl, Hb 7-9 g/dl and Hb > 9 g/dl. In each one of these groups we collected data about transfusion, vasospasm, GOS at 1 month, and ranking at 3 months and one year.

## Results

319 patients were included, 189 male (60%), 62% of them smoked and 41% had high blood pressure (HBP). Clinically, 24% of patients at admittance had neurological deficit and 49% loss of consciousness, with GCS < 7 in 24% of them. 23'5% had level III in Fisher scale and 56% were Fisher IV. Arteriography was performed in 90% of the patients included. Endovascular treatment was done in 52% versus 13% that needed surgical treatment. As complications during admittance, patients developed vasospasm in 35% of the cases (27% before 7 days) and stroke 23% of them. Intracranial hypertension was diagnosed in 38%, and global mortality the first week was 12%. About 1'6% of patients included had hemoglobin levels < 7 g/dl, and all of them recieved transfusion support; 80% had stable levels of Hb above 9 g/dl and they were not transfused in any case. 18'2% had levels between 7 and 9 g/dl, of whom 48% recieved transfusion support. No statistically significant differences were observed when comparing patients transfused with Hb between 7-9 g/dl, with the group who did not recieved transfusion with same levels of Hb, in terms of vasospasm incidence (41% vs 48%), GOS at 1 month and Rankin at 3 months and 1 year.

## Conclusions

Patients diagnosed of aneurysmatic SAH with hemoglobin levels between 7 and 9 g/dl, who are given transfusion support with red blood cells, seem to have no benefit in terms of complications and prognosis, when compared with the ones not transfused.

